# Healing Efficacy of an EGF Impregnated Triple Gel Based Wound Dressing: In Vitro and In Vivo Studies

**DOI:** 10.1155/2014/493732

**Published:** 2014-07-08

**Authors:** Najmeh Khanbanha, Fatemeh Atyabi, Azade Taheri, Fatemeh Talaie, Mirgholamreza Mahbod, Rassoul Dinarvand

**Affiliations:** ^1^Department of Pharmaceutics, Faculty of Pharmacy, Tehran University of Medical Sciences, P.O. Box 14155-6451, Tehran, Iran; ^2^Nanotechnology Research Center, Faculty of Pharmacy, Tehran University of Medical Sciences, P.O. Box 14155-6451, Tehran, Iran; ^3^Noor Ophthalmology Research Center, Noor Eye Hospital, P.O. Box 19393-3475, Tehran, Iran

## Abstract

To accomplish an ideal wound healing process which promotes healthy tissue growth with less scaring, a novel gel based topical drug delivery system composed of 3 different polymers chitosan, dextran sulfate, and polyvinylpyrrolidone K30 (CDP) was prepared. The physicochemical properties of the prepared gels were investigated in vitro. Gels showed a maximum swelling ratio of 50 ± 1.95 times of dried gel in PBS at pH 7.4. The swelling ratios increase in acidic and alkaline pH to 55.3 ± 1.75 and 65.5 ± 2.42, respectively. In the rheological test, prepared gels revealed viscoelastic properties and a small linear viscoelastic region of 0.166%. In vivo wound healing promoting activities of CDP gels containing 20 *μ*g/mL EGF were evaluated on surgically induced dermal wounds in rats using pathologic examination. The application of CDP gel with incorporated EGF significantly reduced the defect on the rat's skin and enhanced epithelial healing compared with the topical application of the EGF-free CDP gel. The results clearly substantiate the beneficial effects of the topical application of CDP containing EGF in the acceleration of healthy wound healing process with less scarring.

## 1. Introduction

As a fundamental response to tissue injury, wound healing is a normal complex process including four general phases of hemostasis (clot formation), inflammation, cell proliferation, extracellular matrix production, and remodeling, which usually in each phase occurs consequently in a regulated manner [1, 2]. The clot and the surrounding wound tissue secrete a variety of cytokines and growth factors such as platelet derived growth factor (PDGF) and epidermal growth factor (EGF) that simulate wound repair process [3, 4]. Several studies showed that topical use of growth factors such as EGF on the wound could accelerate the rate of the epidermal regeneration and reepithelialization of chronic wounds [5, 6]. To achieve an appropriate efficacy of EGF therapy in wound repair, the local bioavailability of the EGF should be prolonged using a suitable drug delivery system. To increase the low bioavailability of peptides and proteins such as EGF in conventional topical solutions, viscous drug delivery systems such as sponge have been explored [7]. Gel based natural polymers such as chitosan and dextran are used for treatment of dermal wounds [8]. Wound dressing prepared with natural polymers is found to prolong the retention time of therapeutic peptides on the wound and increase the therapeutic effects of these peptides [9]. Dextrans are polysaccharides which are found in bacterial extracellular matrix [10], have a good solubility level in water, and have been investigated for protein delivery in pharmaceutical studies [11, 12]. Dextran sulfate is a negatively charged polysaccharide which is derived from dextran via sulfation and was shown to have appropriate characteristics for the treatment of wounds [13]. Chitosan is a natural polymer derived from chitin, known to stimulate cell proliferation and wound healing process through its hemostatic properties [14]. Moreover bacteriostatic properties of chitosan could be useful in relation to wound treatment [14]. Chitosan and its derivatives have been used successfully for controlled delivery of drugs, peptides, and proteins [15–20]. Wound dressing prepared using chitosan and dextran could absorb the wound exudates and hold the moisture providing a suitable moisturized environment at the wound site [21]. But the electrostatic interaction between dextran and chitosan could reduce the stability of dextran-chitosan gels. We used polyvinylpyrrolidone K30 (PVP K30) as suitable polymer to reduce the dextran-chitosan instability (CDP gel). In this work, we prepared CDP gel as a suitable gel based drug delivery system to increase the bioavailability of therapeutic peptides and proteins such as EGF to wound tissue. Then, we impregnated EGF in CDP gel. EGF impregnated CDP gel was used as suitable wound dressing to accelerate and facilitate wound healing process. Physical properties of wound dressing and the effects of EGF on healing process in comparison with controls were studied in full thickness excision wound model in rats in vivo.

## 2. Material and Methods

### 2.1. Materials

Already characterized low and medium molecular weight chitosan samples (deacetylation degree approximately 95%) were purchased from Chitoclear (Siglujordur, Iceland). Dextran sulfate (MW 8 and 10 kDa) was purchased from Sigma-Aldrich (St. Louis, MO, USA). Polyvinylpyrrolidone K30 (PVP K30) was purchased from BASF (Ludwigshafen, Germany). Recombinant human epidermal growth factor (rhEGF) was obtained from Sichuan Huamai Technology Co (Sichuan, China). All other reagents used were of analytical grade.

### 2.2. Preparation of Gels

#### 2.2.1. Preparation of Chitosan Solutions

Chitosan solutions of low and medium molecular weight were prepared in dilute acetic acid (1% w/w) and the mixtures were heated and stirred at 50°C for 24 h until clear solutions were achieved. Chitosan solutions were neutralized with 1 N solution of NaOH to pH 5.1 and then were degassed at 25°C for 15 min using a sonicator (Elmasonic Elma Hans Schmidbauer GmbH & Co. KG) to remove air bubbles.

#### 2.2.2. Preparation of Dextran-PVP Solution

Dextran sulfate (MW 8 and 10 kDa) was dissolved in deionized water. Known amount of PVP K30 was added to the above solution to obtain dextran-PVP solution.

#### 2.2.3. Preparation of CDP-EGF Gels

For preparation of chitosan-dextran-PVP (CPD) gels, freshly prepared chitosan solution was added dropwise to dextran-PVP solution under slow stirring to achieve the final composition of gel formulations as given in Table 1. For preparation of CDP-EGF gels, EGF was added to prepared CDP gels to obtain a concentration of 20 *μ*g/mL.

### 2.3. Swelling Study

Swelling of gels was studied by a gravimetric procedure. CDP gels were cast into 13 diameter × 4.5 height (approximately 0.59 mm^3^) molds and then vacuum dried at 60°C for 12 h. Dried gels were accurately weighted and swollen in PBS pH 7.4 at 25°C. The swollen gels were removed from the buffers at certain times (5, 10, 20, 30, 45, 60, and 120 min), then blotted to remove excess water, and reweighed. Each experiment was performed in triplicate. The swelling ratio was calculated using the following equation:

(1) $$\begin{array}{c} \text{SR}=\frac{W_{s}}{W_{d}}, \end{array}$$

where *W*_*s*_ is the weight of the swollen gel and *W*_*d*_ is the weight of the dried gel.

To characterize the swelling behavior of the gels the following experiments were conducted. To determine the effect of pH on the swelling ratio of gels, CDP gels were immersed in acetate buffer pH 4.5 and phosphate buffer pH 9 at 25°C for 5, 10, 20, 30, 45, 60, and 120 min. The swelling ratio was calculated as mentioned above. The effect of ionic strength on the swelling ratio of gels was measured in 1 N PBS pH 7.4, 4 N PBS pH 7.4, and 8 N PBS pH 7.4 at 25°C for 5, 10, 20, 30, 45, 60, and 120 min, as mentioned above.

#### 2.3.1. Gel Fraction

Different formulations of gels were cast into 0.59 mm^3^ molds and then vacuum dried at 50°C for 12 h until a constant weight was reached (*W*_*d*_). The dried gels were immersed in 40 mL distilled water at 50°C for 12 h under vacuum. The sol part of immersed gels was extracted from the water. The remaining part was vacuum dried at 50°C for 12 h till constant weight was achieved (*W*_*r*_). The gel fraction percentage was determined using the following equation:

(2) $$\begin{array}{c} \text{Gel}\;\text{Fraction}\;\left(\%\right)=\left(\frac{W_{r}}{W_{d}}\right)\times 100. \end{array}$$

### 2.4. Differential Scanning Calorimetry (DSC) Analysis

The physical or chemical interaction between polymers was analyzed by differential scanning calorimetry (DSC) studies. Samples were analyzed using a differential scanning calorimetric (DSC) analyzer (DSC 823, Mettler-Toledo, Germany). 5 mg of the mixture of chitosan and dextran sulfate or chitosan-dextran sulfate dried gels were placed in aluminum pans. For thermogram acquisition, the samples were heated over a range of 30°C to 500°C at a rate of 10°C per min and the thermal behavior of samples was recorded.

#### 2.4.1. Rheological Studies

To study the plastic deformation stress and the viscoelastic properties of the gel, the rheological analysis of the prepared gels was performed using a stress controlled Paar Physica MCR 300 (Anton Paar GmbH, Austria) with a plate-plate measurement system PP25 (the gap between the plates was 1 mm). The temperature was 25°C for all tests. The data were collected using Rheoplus/32 Service V3.40 software (Anton Paar GmbH, Austria). Gels were placed between the plates of the measuring system and two sets of experiments were performed.

The amplitude sweep test (oscillatory condition) was performed (25°C) in the range of 0.01–100 Pa for the gels. The storage modulus (*G*′), the loss modulus (*G*′′), and the linear viscoelastic (LVE) deformation range were investigated using this test.

Further, gel flow measurement was performed using a plate-plate measurement system PP25 (the gap between the plates was 1 mm) with a shear stress range of 1–100 Pa. The yield point of the gel was determined.

#### 2.4.2. In Vivo Studies

All in vivo experiments were conducted according to the guidelines of Ethical Committee of the Pharmaceutical Research Centre, Faculty of Pharmacy, Tehran University of Medical Sciences, Iran. 15 male Wistar rats (275 ± 25 g) were purchased from Pasteur Institute (Tehran, Iran). Animals had free access to food and water. The animals were divided into three groups. There were 5 animals in each group. Rats' dorsal hair was depilated while the animals were under ketamine/xylazine anesthesia. Skin tissue (1.5 cm long × 1.5 cm) was surgically removed using sterile surgical tools to create full thickness wound on the back of the animal. The animals were housed individually under standardized environmental conditions (day 0). In first group, 0.4 mL of CDP gel containing 20 *μ*g/mL EGF was locally applied to the full thickness skin wounds, from day 1, once a day for 11 days. The second group received the same CDP gel formulation without EGF and the third group was the control group in which the animal was wounded but no treatment was applied.

Before using CDP gel with EGF (20 *μ*g/mL), all the animals' wounds in all groups were washed with sterile normal saline solution and photographed and then the gels were applied on the wounds. Wounds were covered with nonwoven retention tape using adhesive 3M tape. After 8 experimental days, three rats of each group were sacrificed by chloroform inhalation. Other remaining animals were sacrificed after 11 experimental days. The images of wounds were captured after rising of wounds with normal saline using a digital camera. The images of wounds were analyzed for the wound area using Photoshop (versus CS5) software. Tissue specimens from the wound site of individual rats were excised after animal euthanization. The skin samples were fixed in 10% formaldehyde solution, dehydrated with graded alcohol, cleared in xylene, and then embedded in paraffin block. 5 *μ*m sections have been cut from blocks, stained with Haematoxylin and Eosin, and observed under a light microscope.

#### 2.4.3. Statistical Analysis

Statistical significance was determined by a one way analysis of variance (ANOVA) followed by a post hoc test for multiple comparisons using SPSS Vr.3 software. *P* < 0.05 was considered statistically significant.

## 3. Results

### 3.1. Preparation of CDP Gels

The viscosity of 3% (and higher) of chitosan solutions was too high and thus these solutions were not suitable for gel preparation process. Moreover the viscosity of 1% (and lower) of chitosan solutions was too low and thus these solutions were not suitable for gel preparation process. Thus, chitosan solutions with concentration of 2% w/w were selected for gel preparation. For prevention of the formation of chitosan-dextran precipitation (because of electrostatic interaction), PVP K30 was used as a bulking polymer to slow down the electrostatic interaction and precipitation.

### 3.2. Swelling Behavior of CDP Gels

**Figure 1:**
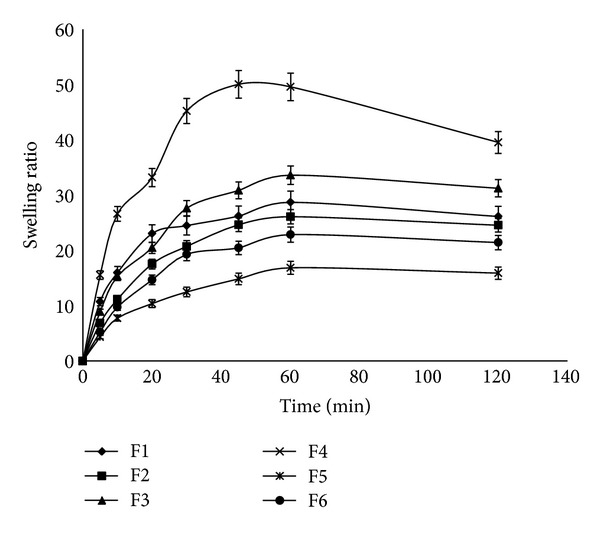
Measurement of swelling ratio obtained from different chitosan-dextran-PVP gels presented in Table 1 in PBS (pH 7.4) at 25°C at predetermined time points (5, 10, 20, 30, 45, 60, and 120 min) (*n* = 3, mean ± SD).

**Figure 2:**
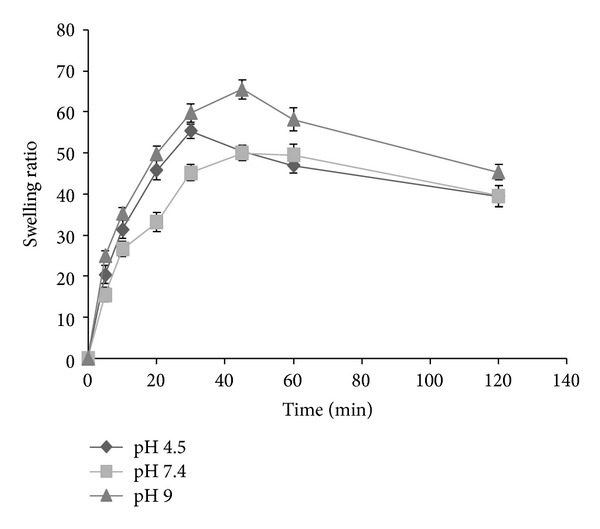
Measurement of swelling ratio of chitosan-dextran-PVP gel (F4) in acetate buffer (pH 4.5), PBS (pH 7.4), and phosphate buffer (pH 9) at 25°C at predetermined time points (*n* = 3, mean ± SD).

**Figure 3:**
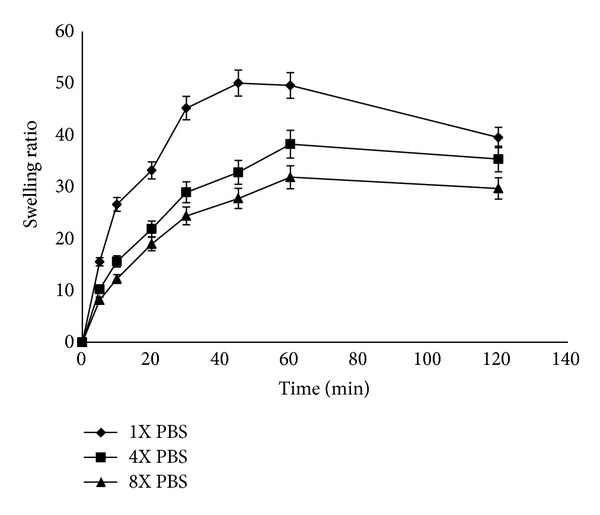
Measurement of swelling ratio of chitosan-dextran-PVP gel (F4) in 1 N PBS (pH 7.4), 4 N PBS (pH 7.4), and 8 N PBS (pH 7.4) at 25°C (*n* = 3, mean ± SD).

Water uptake of CDP gels was followed gravimetrically. The swelling curves of CDP gels in PBS pH of 7.4 at 25°C are given in Figure 1. CDP gels in PBS at pH 7.4 show high swelling ratios from 16.84 ± 1.95 for F5 to 50.03 ± 1.06 for F4. F4 showed the highest swelling ratio (50.03) in PBS at pH 7.4 after 45 minutes. Thus, F4 containing 1.6% low molecular weight chitosan, 0.2% dextran sulfate, and 0.2% PVP was chosen as an optimum sample to conduct further experiments. Swelling behavior was carried out for F4 at different pH values (4.5, 7.4, and 9) (Figure 2). Maximum swelling ratio of F4 was observed at pH 9.0 whereas lesser but significantly different swelling ratio was observed in other pH values in this study. Effect of ionic strength on the swelling ratio of the candidate gel (F4) was investigated at different concentration of PBS (pH 7.4) at 25°C (Figure 3). It was observed that as the concentration of PBS (pH 7.4) increased (ionic strength increased consequently), a decrease in gel swelling ratio occurred.

### 3.3. Gel Fraction

**Figure 4:**
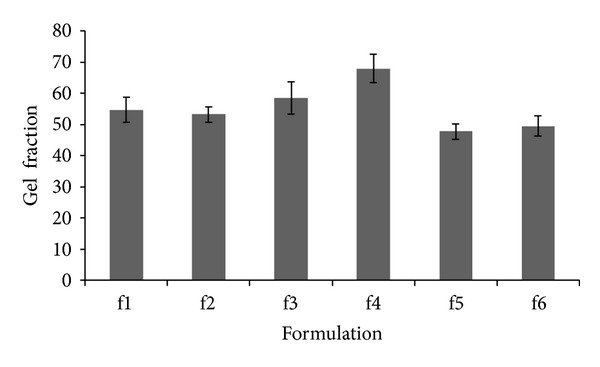
Gel fraction (%) of chitosan, dextran, and PVP gels.

The gel fractions of F1, F2, F3, F4, F5, and F6 CDP gel formulations were 54.74%, 53.21%, 58.56%, 67.92%, 47.76%, and 49.50%, respectively. F4 showed the highest gel fraction among other formulations (Figure 4). Thus, according to the results of swelling ratio and gel fraction studies, F4 formulation was chosen to perform further studies.

### 3.4. DSC Analysis

**Figure 5:**
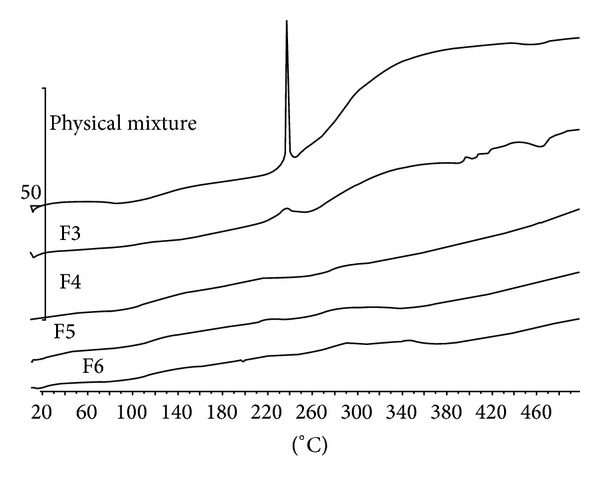
DSC thermogram of physical mixture of chitosan-dextran (F3, F4, F5, and F6).

The differential scanning calorimetry (DSC) thermograms of CDP gels are shown in Figure 5. An exothermic peak was observed in DSC thermogram of physical mixture of chitosan, dextran, and PVP at about 220–240°C. The thermograms of gels gave no intrinsic peak but showed an approximately similar pattern to thermogram of physical mixture of chitosan, dextran, and PVP (Figure 5).

### 3.5. Rheological Properties

**Figure 6:**
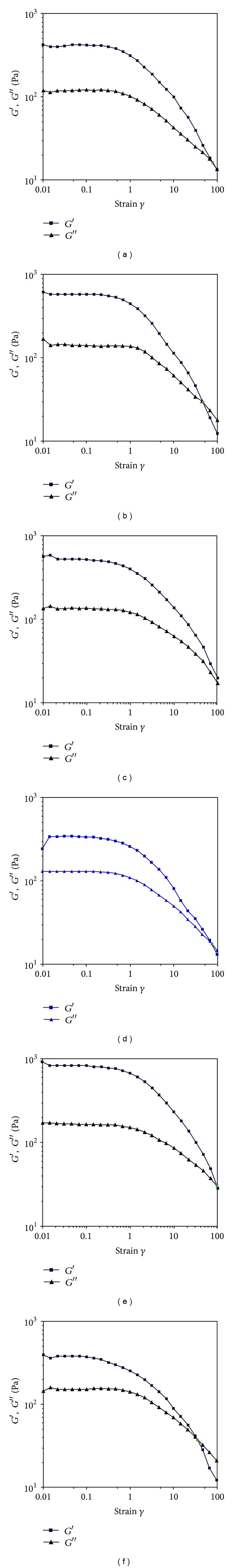
The strain sweep test of gel: (a) F1, (b) F2, (c) F3, (d) F4, (e) F5, (f) F6.

The strain sweep test shows large differences between the elastic *G*′ and the loss *G*′′ moduli, with *G*′ much higher than *G*′′ indicating higher viscoelastic properties of the prepared gel as shown in Figure 6. However the rapid decrease in modulus resulted in a very small LVR (linear viscoelastic region). Flow measurement was conducted to determine the plastic deformation stress, that is, the yield point of gels (Table 2). The portion of *G*′′ to *G*′ is defined as tangent *δ*, angle from 0 degrees indicating an absolute elastic gel to 90 degrees indicating an absolute viscous gel. The results show that as the strain increases, *G*′ decreases more rapidly than *G*′′ illustrating a more elastic gel transforms into a more viscous gel. Consider

(3) $$\begin{array}{c} \frac{G^{\prime \prime }}{G^{\prime }}=\tan\delta . \end{array}$$

### 3.6. In Vivo Wound Healing Study

**Figure 7:**
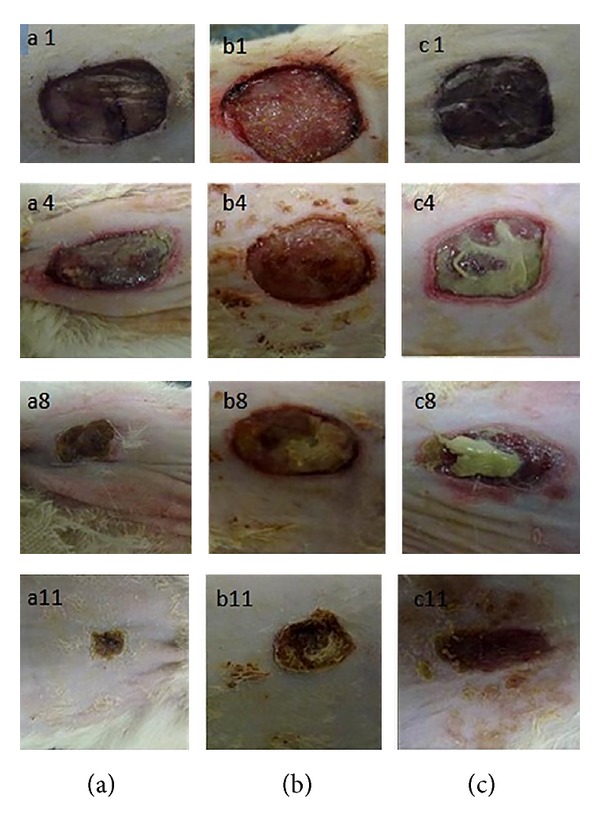
Photographs of the relative size reduction of the wounds in rat. (a) Wound treated with CDP gel containing EGF, (b) wound treated with CDP gel without EGF, and (c) untreated group (control group) on days 1, 4, 8, and 11 after operation (*n* = 3, mean ± SD).

**Figure 8:**
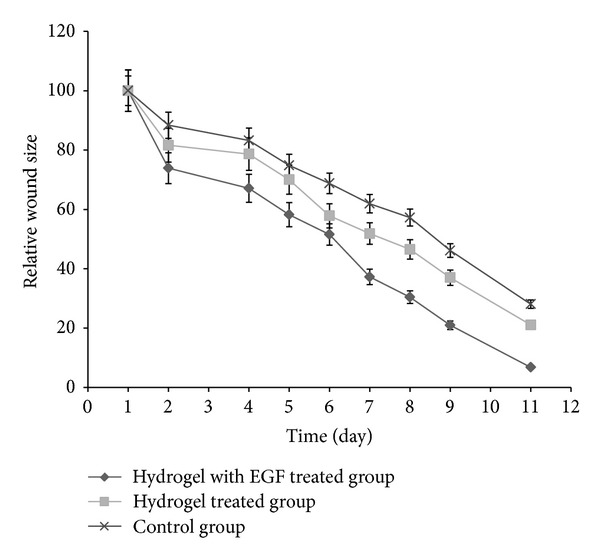
The relative size reduction of the wounds treated with CDP gel with EGF, CDP gel without EGF, and untreated wounds (*n* = 3, mean ± SD).

According to data obtained from gel fraction and swelling studies, F4 formulation was chosen for the treatment of full thickness wounds on rats. Macroscopic appearance of wounds treated with F4 formulation of CDP gel with EGF, F4 formulation of CDP gel without EGF and untreated group (control group) are shown in Figure 7. Each wound was observed for a period of 1, 4, 8, and 11 days after operation. All rats survived in the postoperative period until sacrifice. There were no evidences of necrosis. The formulation of CDP gels with and without EGF induced no infection of the wounds, whereas the control group showed wound infection at 4 and 8 days after operation. The relative size reduction of the wounds treated with CDP gel with EGF, CDP gel without EGF, and untreated wounds is illustrated in Figure 8. At 8 and 11 days after operation, a significant size reduction in the wounds treated with CDP gel with EGF compared to CDP gel without EGF treated groups and control group was observed. Furthermore, the CDP gels with EGF gave about 90–95% of wound size reduction at 11 days after operation.

## 4. Histological Examination

**Figure 9:**
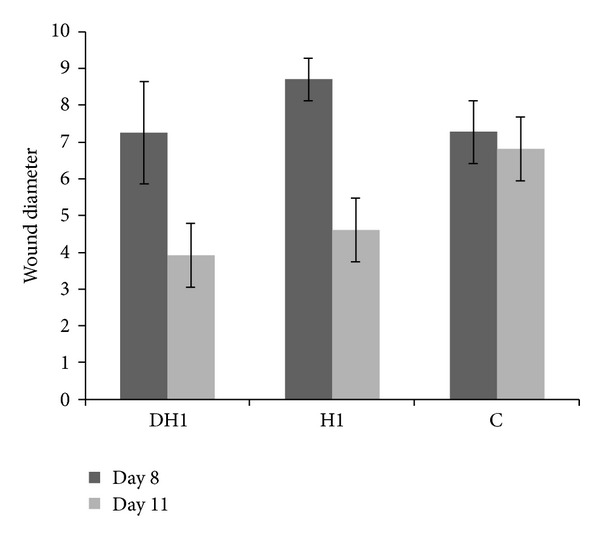
Histopathological wound diameter evaluation of the wounds treated with CDP gel with EGF (DH1), CDP gel without EGF (H1), and untreated wounds (c).

As shown in Figure 9, at the end of day 8, there is not any significant difference in diameter of ulcers in different groups of study (*P* ≥ 0.05). However, the group treated by CDP gel with EGF reveals the highest rate of new epithelium formation. The greatest diameter of ulcer in CDP gel with EGF treated group at day 11 (3.92 ± 1.6) was significantly lesser than CDP gel without EGF treated group (4.6 ± 1.32) and control group (6.8 ± 1.8) (*P* ≤ 0.05).

**Figure 10:**
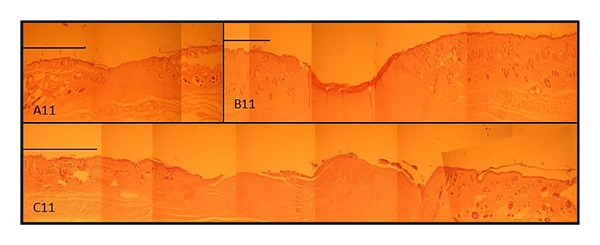
Photographs of treated wound areas. (A11) wound treated with CDP gel containing EGF, (B11) wound treated with CDP gels without EGF, and (C11) untreated group (control group) on day 11 after operation. Scale bar: 1 mm.

**Figure 11:**
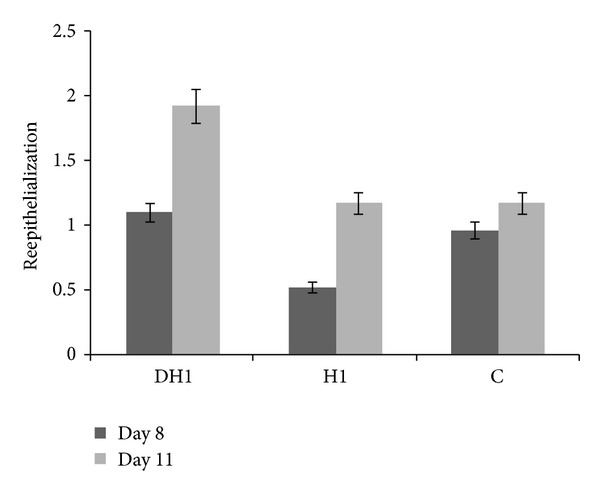
Histopathological reepithelialization diameter evaluation of the wounds treated with CDP gel containing EGF (DH1), CDP gel without EGF (H1), and untreated wounds.

The reepithelialization diameter of the groups (Figure 11) treated with gels containing EGF was 1.1 ± 0.65 and 1.925 ± 1.53 at 8 and 11 days after operation, while the control group revealed 0.96 ± 0.2 and 1.175 ± 0.55 cm at 8 and 11 days after operation. In days 8 and 11 of reepithelialization improvement in diameter in group treated with CDP gel containing EGF was highest (not statistically significant). The microscopic photos of the wounds at day 11 are shown in Figure 10. Wound epithelium thickness was of quite similar values in all groups (approximately 150 *μ*m), data not shown.

## 5. Discussion

In this study, a novel drug delivery system composed of a combination of three gels carrying EGF was prepared, characterized, and studied in vivo. Our experiments show that CDP gel could be used as a novel improved carrier for EGF. EGF could induce the wound healing process and minimize the scar formation. EGF could induce cell proliferation and accelerate wound healing process and skin repair through regulation of extracellular matrix proliferation and epidermal stem cells differentiation. Thus, the local increase in concentration of EGF at the wound site could accelerate the wound healing process [22, 23]. Following trauma the level of endogenous EGF increases to accelerate tissue repair process, but the amount of endogenous EGF is not sufficient for suitable wound repair. Thus, the application of exogenous EGF on the wound site could accelerate the wound healing process [24, 25]. Tanaka et al. showed that the continuous use of EGF is a necessary factor for maximum wound healing [25]. Moreover they demonstrated that the use of EGF in gel formulations was more effective for wound healing promotion than the use of EGF in solutions. The gel formulations could remain on the wound surface for a long time and provide a suitable concentration of EGF on the wounded area, while solutions have fast drainage [26]. Moreover, gel based wound dressings are suitable formulations for the treatment of wounds and burns because they absorb the exudates of wounds and maintain suitable moisture on the wound surface [27, 28]. In practice suitable gel based wound dressings could be applied easily [29, 30] and could be removed easily since the skin is injured [31]. In the present novel drug delivery system, we were able to take advantage of the different properties of different polymers (chitosan and dextran) in wound healing while preventing instability due to electrostatic interactions between chitosan and dextran polymers. A mixture of chitosan and dextran sulfate forms a gel at room temperature but electrostatic interactions between amino groups of chitosan and sulfate groups of dextran sulfate could cause the formation of chitosan-dextran precipitation. To prevent formation of chitosan-dextran sulfate precipitate, PVP was used to decrease the chitosan and dextran interactions. Similar pattern of the DSC thermograms obtained from CDP gel and chitosan, dextran, and PVP gels suggests that no chemical modification occurs in CDP gel [32]. The presence of PVP is probably also preventing a potential gel destabilization due to the change in ratio of the two other components (chitosan and dextran) in the final gel which could likely leave one of the components in excess with respect to the other. Moreover, EGF has a negative charge at pH above 4.6 (isoelectric pH of EGF) [33]; thus, the electrostatic interaction between negatively charged EGF and amino groups of chitosan could also sufficiently entrap EGF in the CDP gel.

In the present study the results obtained from gel fraction studies show that F4 CDP gel formulation has the highest gel fraction. An ideal wound dressing should be able to absorb the exudates of wound and decrease the risk of microbial infection that could initiate through the increased accumulation of wound exudates. Moreover, a suitable wound dressing should maintain the moisture on the surface of the wound during treatment to prevent scabbing. The conventional wound dressings absorb the wound moisture and fully dry up the damaged site causing the bandaging to stick to the wound, leading to further damage. Therefore, the swelling behavior and absorption capacity of gels could be important criteria in the design of a suitable gel based wound dressing. The high swelling ratio of F4 formulation which could in part be contributed to lower percentage of PVP as compared to F1 formulation shows that this CDP gel could provide an adequate drainage of wound exudates and suitable levels of moisture on the wound surface. Moreover, the combinatory antimicrobial effects of chitosan and dextran could protect the wound from the external contamination. Thus, CDP gel has different complimentary properties that make it suitable for promoting wound healing. Generally, as the gel fraction of gel increases, the strength of the gel increases [34, 35] which makes F4 formulation a suitable gel for in vivo studies.

The prepared gel showed appropriate rheological properties. In strain sweep tests, large differences were observed between the elastic *G*′ and the loss *G*′′ moduli, with *G*′ being much higher than *G*′′, which could indicate the higher viscoelastic properties of the prepared gel. A small viscoelastic “plateau” and a rapid decrease in modulus resulting in a very small LVR (linear viscoelastic region) indicate that the structure is a “weak gel” and ready to flow at higher strain [36, 37]. The CDP gel revealed viscoelastic properties with a small LVR (linear viscoelastic region) of 0.166%. This nonlinear rheological behavior can be seen in gels based on physical interaction (e.g., hydrogen bond, ionic interaction, and entanglement of polymeric chain). This in turn could be contributed to partial and not complete cross linking that allows the sliding movement of polymeric domains over each other [38, 39] and indeed this property seems to be important due to the nature of the skin injury. Flow measurement was conducted to determine the plastic deformation stress, that is, the yield point of gels. The higher the yield point is, the more deforming resistant the gel is. Although all prepared gels are found to have physical and weak ionic interaction, F4 prepared with low molecular weight chitosan (1.6%), 0.2% dextran sulfate (10 KD MW), and 0.2% of PVP has a higher yield point around 64 Pa and is therefore more resistant to deformation. Mechanical properties of gels are very important for pharmaceutical applications. For example, maintaining the integrity of a drug delivery device during the lifetime of the application is crucial.

The results of our study showed a significant promotion in the wound healing process in the group given CDP gel containing EGF treatment, compared to treatment with the same gel without EGF and control group. Furthermore, pathological studies showed that epidermis length had also increased significantly in the CDP gel containing EGF treated group, compared to CDP gel treated group or control group. The results indicate that CDP gel combined with EGF could significantly improve epidermal cell proliferation and differentiation, thereby promoting wound healing. It is though important to note that in our experiments the control group showed infection, a phenomenon that can lead to delay in the healing process. In healing process of skin wounds, both epithelial and mesenchymal cells (fibroblasts) are involved. Fibroblasts are in charge of repair of dermis and they proliferate and release extracellular matrix. These two lead to repair of dermis with keloid formation and resultant contracture of wound. On the other hand, epithelial cells proliferate and cover the keloid to make a complete repair. If they fail to cover all the surface of keloid, scar of wound would remain. On the other hand, animals receiving the EGF-free F4 gel showed less infection which might be due to the higher percentage of chitosan; EGF especially might be reacting with polyelectrolytes and thus further increasing the amount of available chitosan, fortifying the antibacterial properties. Finally, CDP gels containing EGF accelerated the wound healing process in vivo. Prepared gels could be easily applied on the wound and removed from the site. Moreover rising the concentration of EGF at the wound site following application of the gels could promote/facilitate wound healing process with a low chance of scar formation.

## 6. Conclusions

In this study chitosan-dextran-PVP gel (CDP gel) prepared as a novel and suitable gel based drug delivery system intended to increase the bioavailability of therapeutic peptides and proteins such as EGF toward wound tissue. Wound dressing prepared using chitosan and dextran could absorb the wound exudates and hold the moisture providing a suitable moisturized environment at the wound site. But the electrostatic interaction between dextran and chitosan could reduce the stability of dextran-chitosan gels. In comparison with other marketed gel based wound dressing products prepared with chitosan and dextran, in this study polyvinylpyrrolidone K30 (PVP K30) was applied as a suitable polymer to reduce the dextran-chitosan instability (CDP gel). CDP gels containing EGF accelerated the wound healing process in vivo while easily applied to or removed from the wound. As a conclusion, the present gel can be applied as a suitable wound dressing to increase EGF concentration at wound site and to promote/facilitate wound healing with a low chance of scar formation.

## Figures and Tables

**Table 1:** Composition of CDP gel formulations.

Samples	Concentration %				
	Low molecular weight chitosan	Medium molecular weight chitosan	Dextran sulfate (MW 8 kDa)	Dextran sulfate (MW 10 kDa)	PVP
F1	1.33%		0.33%		0.33%
F2	1.5%		0.25%		0.25%
F3	1.6%		0.2%		0.2%
F4	1.6%			0.2%	0.2%
F5		1.6%	0.2%		0.2%
F6		1.6%		0.2%	0.2%

**Table 2:** Rheological properties of gel.

Oscillatory test												Flow test
	Flow point			*G*′		*G*′′		tan⁡*δ*		Angle		Yield point
	Stress	Strain	Crossover	% strain								Shear stress
				0.01	100	0.01	100	0.01	100	0.01	100	
F1	18.0	79	16.2	423	13	118	14	0.3	1.1	15.6	47.1	38.6 Pa
F2	19.2	47	28.9	614	12.3	166	17.5	0.3	1.4	15.1	54.9	38.9 Pa
F3	No	No	No	569	20	134	17	0.24	0.9	13.3	40.7	No
F4	18.8	75.8	17.5	245	13	129	14.6	0.5	1.1	27.8	48.1	64.2 Pa
F5	41.3	96	30.3	916	28.6	168	29.6	0.2	1	10.4	46	67.6 Pa
F6	18.7	35.6	37	386	12.1	143	21.1	0.4	1.7	20.3	60.2	37.4 Pa

## Abbreviations

PVP K30: Polyvinylpyrrolidone K30
CDP gels: Chitosan, dextran sulfate, and polyvinylpyrrolidone K30 gels
PDGF: Platelet derived growth factor
EGF: Epidermal growth factor
rhEGF: Recombinant human epidermal growth factor.
